# Gender- and age-related differences in the width of attached gingiva and clinical crown length in anterior teeth

**DOI:** 10.1186/s12903-021-01639-4

**Published:** 2021-06-04

**Authors:** Marie-Elise Jennes, Claudia Sachse, Tabea Flügge, Saskia Preissner, Max Heiland, Susanne Nahles

**Affiliations:** 1 Department of Prosthodontics, Geriatric Dentistry and Craniomandibular Disorders, Charité – Universitätsmedizin Berlin, Corporate Member of Freie Universität Berlin, Humboldt-Universität Zu Berlin, Assmanhauser Straße 4-6,, 14197 Berlin, Germany; 2 Department of Oral and Maxillofacial Surgery, Charité – Universitätsmedizin Berlin, Corporate Member of Freie Universität Berlin and Humboldt Universität Zu Berlin, Augustenburger Platz 1, 13353 Berlin, Germany; 3 Department of Oral and Maxillofacial Surgery, Charité – Universitätsmedizin Berlin, Corporate Member of Freie Universität Berlin and Humboldt Universität Zu Berlin, Hindenburgdamm 30, 12203 Berlin, Germany

**Keywords:** Gender-related changes, Age-related changes, Attached gingiva, Crown length

## Abstract

**Background:**

The anatomical features of the gingiva and the clinical crowns and their interrelation, especially in aesthetically and functionally demanding areas, are important in complex dental or implant-retained prosthetic rehabilitations. This observational cross-sectional study was designed to evaluate gender- and age-related differences in the width of attached gingiva (WAG), the clinical crown length (CCL), and their interrelation in the anterior teeth to determine the relationship between the pink and white aesthetics.

**Methods:**

Eighty (54 females, 26 males) fully dentate Caucasian participants between the ages of 20 and 25 years and 36 probands (23 females, 13 males) between the ages of 45 and 55 years were included in the present study. The CCL of the maxillary and mandibular canines, as well as the central incisors of the maxilla and mandible, were determined with a dental sliding caliper measuring from the middle margin of the gingiva at its deepest point to the incisal edge. The clinical investigation of the WAG was performed by inserting a periodontal probe into the gingival sulcus in the middle of the buccal surface to firstly measure the probing pocket depth. The distance between the gingival margin and mucogingival junction (MGJ) was then measured with a Beerendonk sliding caliper in the middle of the labial curvature, and the clinical WAG was determined by subtraction of the measured probing depth. For the statistical analysis, the Mann–Whitney *U* test, the Wilcoxon-Test, the Spearman’s rank correlation, and a two-factorial non-parametric analysis were used.

**Results:**

There was no correlation between the CCL and the WAG in a healthy periodontium. Gender influenced the CCL, with men having significantly longer teeth than women in both maxilla (*P* ≤ 0.01) and mandible (*P* ≤ 0.05). Age did not influence the CCL significantly neither in the upper (*P* = 0.06) nor in the lower jaw (*P* = 0.33). Gender did not show to have a significant influence on the WAG of maxilla (*P* = 0.69) and mandible (*P* = 0.26). But differences in the WAG between young and old participants were observed in both upper (*P* ≤ 0.01) and lower jaw (*P* ≤ 0.05).

**Conclusion:**

The present observational study demonstrated that the mean values of cohorts with mixed age groups and genders should not be considered when attempting to determine the ideal relationships between the pink and white aesthetics since the statistical analyses showed significant differences between different age groups and genders.

## Background

Complex dental or implant-retained prosthetic rehabilitation is one of the biggest challenges in dentistry, especially in aesthetically and functionally demanding areas. An optimum harmonization of the white and pink aesthetics is essential for an aesthetic outcome [1] and is known to influence the social attractiveness of individuals [2]. In this context, the “white aesthetic” describes the natural dentition or the restoration of dental hard tissue with suitable materials, whereas the “pink aesthetic” refers to the soft tissue surrounding the teeth, which includes the gingiva and the interdental papilla [3]. The relevance of the interplay between these two parameters is particularly apparent in a gummy smile with excessive gingival display due to an impaired ratio between the soft tissue and clinical crowns [4].

For treatment planning of the restoration of extended hard and soft tissue defects, knowledge of a favorable ratio and the precise diagnosis of the interrelation of size and shape of the clinical crowns and the involved gingiva are essential. Intra- and inter-individual variations exist, with many features of pink aesthetics are genetically defined and have become the subject of considerable interest from therapeutic and epidemiological points of view [5, 6].

However, studies regarding the white aesthetics and factors influencing them are scarce. Based on published results, the clinical crown length (CCL) is not static during life [7]; however, nothing is known about the age-related inter-individual comparison between the pink and white aesthetics.

The gingiva can be morphologically divided into attached gingiva, which is the most apically located part, up to the mucogingival junction (MGJ), the more coronally located free marginal gingiva, which extends to the free gingival margin and the papillary gingiva [8]. The surface of the attached gingiva is keratinized and is better suited to withstand mechanical irritations than alveolar mucosa, which has a non-keratinized epithelium [9]. However, the width of the alveolar mucosa and attached gingiva varies depending on the region and among individuals [10], and a few studies have shown an increase in the width of attached gingiva (WAG) with age [10–13].

Besides the influence of age on the WAG and CCL, gender may also have an effect; although, reports are contradictory [6, 14]. In an Indian population, the gingiva was wider in females than males [15], while other studies have found no correlation with gender [14]. Regarding the CCL, men have been found to have significantly longer teeth than women [16, 17].

Although there are various studies on populations of different origins and age groups regarding age- and gender-specific differences in the WAG and CCL, to best of our knowledge, no data exist for a single cohort in a Caucasian population.

Thus, the aim of the present observational study was to evaluate gender- and age-related differences in the WAG and the CCL and their interrelation in the anterior teeth to determine the relationship between the pink and white aesthetics.

## Methods

### Participants

The research proposal for this observational cross-sectional study was approved by the Ethics Committee of the Charité Universitätsmedizin Berlin, Germany (EA4/064/18), and was performed in accordance with the Declaration of Helsinki. Informed consent was obtained from each participant.

Eighty (54 females, 26 males) fully dentate probands between the ages of 20 and 25 years and 36 probands (23 females, 13 males) between the ages of 45 and 55 years were included in the present study. All probands were selected from the Caucasian population. Analyses of the CCL and WAG were performed on the following teeth: the maxillary and mandibular canines (13, 23, 33, 43) and the maxillary and mandibular central incisors (11, 21, 31, 41). Since this was an observational cross-sectional study, there was no predetermined sample size. All participants that fulfilled the following criteria were included.

### Inclusion criteria

The inclusion criteria were patients with full natural maxillary and mandibular dentition in neutral occlusion without fillings or prosthetic crowns on the examined teeth, recessions, abrasion, attrition, or signs of gingival inflammation or periodontal disease. For evaluation of inflammatory processes, an inflammation index [18] was used. Participants with a gingival index of ≥ 1 were excluded.

### Clinical parameters and measurement

All measurements were performed three times at three different time points by the same investigator in order to minimize the measurement error during the examination. From this, the arithmetic mean was determined, and the values were calculated. The CCL of the anterior teeth in the upper and lower jaw were measured using a dental Beerendonk sliding caliper (Dental Liga, Cologne, Germany) by measuring from the middle margin of the buccal gingiva at its base to the incisal edge.

**Fig. 1:**
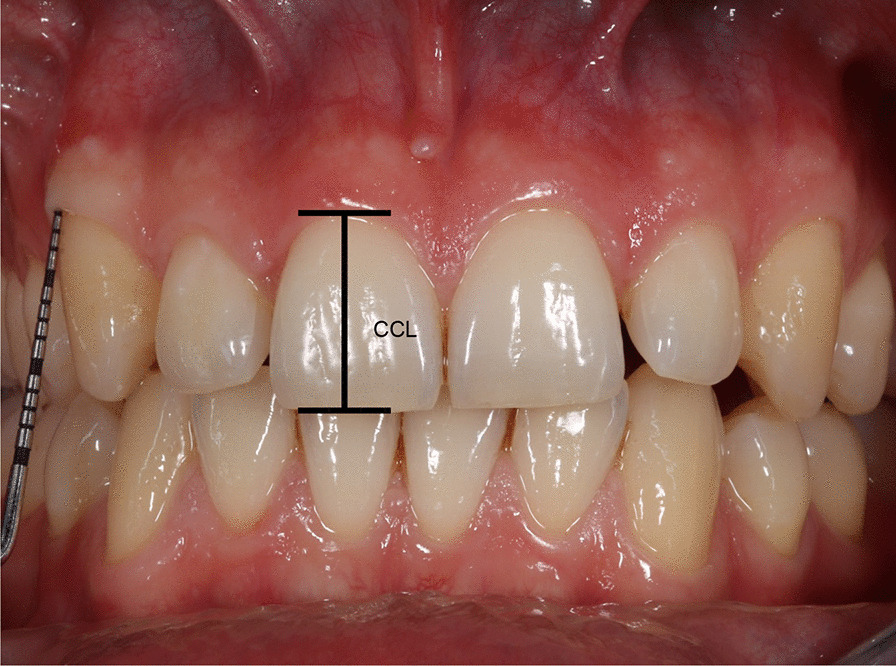
The clinical crown length (CCL) of the anterior teeth in the upper and lower jaw was measured with a dental Beerendonk sliding caliper by measuring from the middle edge margin of the buccal gingiva at its base to the incisal edge. The clinical investigation of the width of attached gingiva (WAG) was performed by inserting a UNC-15 periodontalWHO probe in the middle part of the buccal surface into the gingival sulcus until firm resistance was felt. The distance was than measured between the gingival edge margin and the base of the sulcus

**Fig. 2:**
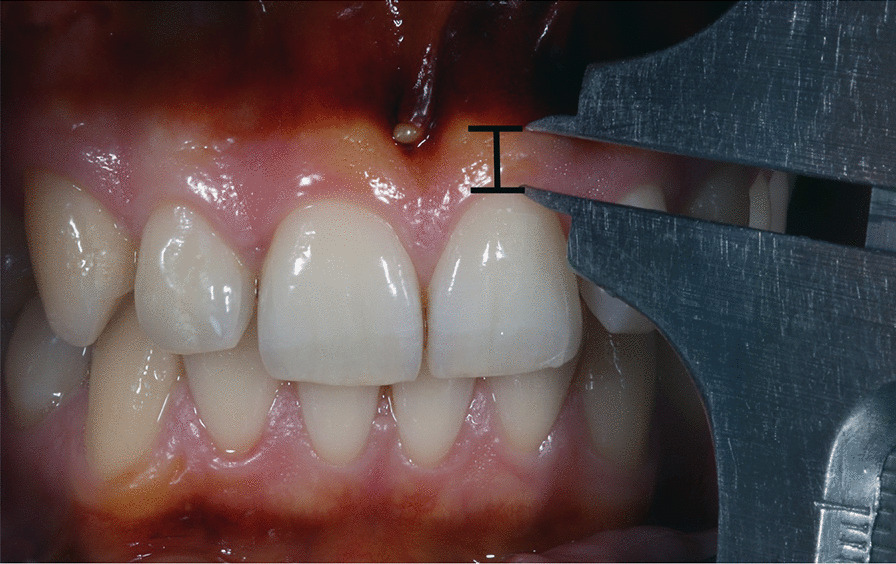
The gingiva was colored with 5% Lugol´s iodine solution, which only stains the alveolar mucosa and clearly demarcates the mucogingival junction. The distance between the gingival edge margin and mucogingival junction (Width of keratinized gingiva) was than measured with a Beerendonk sliding caliper in the middle part of the buccal surface. The width of attached gingiva (WAG) was determined by subtraction of the measured probing depth form the width of keratinized gingiva

The clinical investigation of the WAG was performed by inserting a UNC-15 Periodontal probe into the gingival sulcus in the middle of the buccal surface until firm resistance was felt. The distance between the gingival edge and the base of the sulcus was then measured (Fig. 1). Subsequently, the gingiva was colored with 5% Lugol´s iodine solution (Laborladen, Hüfingen, Germany), which only stains the alveolar mucosa and clearly demarcates the MGJ. The distance between the gingival margin and MGJ (Width of keratinized gingiva) was then measured with a Beerendonk sliding caliper in the middle of the buccal surface. The WAG was determined by subtraction of the measured probing depth from the width of keratinized gingiva (Fig. 2). According to Fischer-Brandies [19] the Beerendonk sliding caliper provides an accuracy of 0.1 mm.

### Statistical analysis

The Mann–Whitney *U* test was performed to analyze differences between gender and age and between the ratios of the quotient CCL:WAG in the maxilla and mandible, whereas the Wilcoxon rank test was used to show differences between the CCL and WAG regarding tooth position. Spearman’s rank correlation coefficient was calculated to assess the relationship between the CCL and WAG. Because of not normally distributed data a two-factorial non-parametric analysis [20] was used to determine the influence of age and gender on the CCL and WAG. Descriptive statistical analysis was performed in SPSS Statistics 23.0 (IBM, Armonk, IL, USA). The results were considered statistically significant at a *P* value ≤ 0.05.

## Results

The main focus was the evaluation of the differences between young and old probands regarding CCL and WAG. The data of the power analysis is described in Table 1.

**Table 1:** Power analysis for CCL and WAG in young and old probands

Two-group Satterthwaite t-test of equal means (unequal variances) (unequal n's)		
	CCL: young vs. old	WAG: young vs. old
Test significance level, α	0.050	0.050
1- or 2-sided test	2	2
Group 1 mean, μ_1_	9.350	2.990
Group 2 mean, μ_2_	9.630	3.430
Difference in means, μ_1_–μ_2_	− 0.280	− 0.440
Group 1 standard deviation, σ_1_	1.240	1.190
Group 2 standard deviation, σ_2_	1.480	1.530
Power (%)	50	86
n_1_	320	320
n_2_	144	144

### CCL

The average CCL of maxillary teeth was 9.9 mm (SD = 1.17 mm) for incisors and 9.7 mm (SD = 1.19 mm) for canines. In the mandible, an average CCL of 8.3 mm (SD = 0.97 mm) was documented for the incisors and 9.8 mm (SD = 1.24 mm) for the canines. The distribution of the CCL with regard to age and gender is displayed in Table 2.

**Table 2:** Distribution of CCL (mm) and WAG (mm) with regard to age and gender

	Clinical Crown Length (CCL)		Width of Attached Gingiva (WAG)	
	Young	Old	Young	Old
*Maxilla*				
N	160	72	160	72
Mean value	9.71	10.05	3.64	4.22
95% CI	9.53–9.88	9.74–10.35	3.47–3.82	3.87–4.57
Minimum	6.9	6.70	1.25	0.30
Maximum	12.05	14.80	6.40	8.00
Standard deviation	1.13	1.29	1.12	1.48
*Mandible*				
N	160	72	160	72
Mean value	8.99	9.22	2.33	2.63
95% CI	8.79–9.18	8.85–9.58	2.19–2.47	2.37–2.89
Minimum	5.05	6.50	0.80	0.30
Maximum	12.20	14.90	5.00	7.90
Standard deviation	1.24	1.55	0.86	1.12

	Female	Male	Female	Male
*Maxilla*				
N	154	78	154	78
Mean value	9.58	10.27	3.77	3.92
95% CI	9.41–9.75	9.97–10.56	3.58–3.96	3.59–4.24
Minimum	6.90	6.70	1.25	0.30
Maximum	12.00	14.80	6.70	8.00
Standard deviation	1.05	1.30	1.19	1.42
*Mandible*				
N	154	78	154	78
Mean value	8.88	9.43	2.37	2.54
95% CI	8.68–9.06	9.08–9.78	2.22–2.50	2.29–2.78
Minimum	5.05	6.50	0.30	0.40
Maximum	11.45	14.90	5.10	7.90
Standard deviation	1.19	1.55	0.89	1.08

**Fig. 3:**
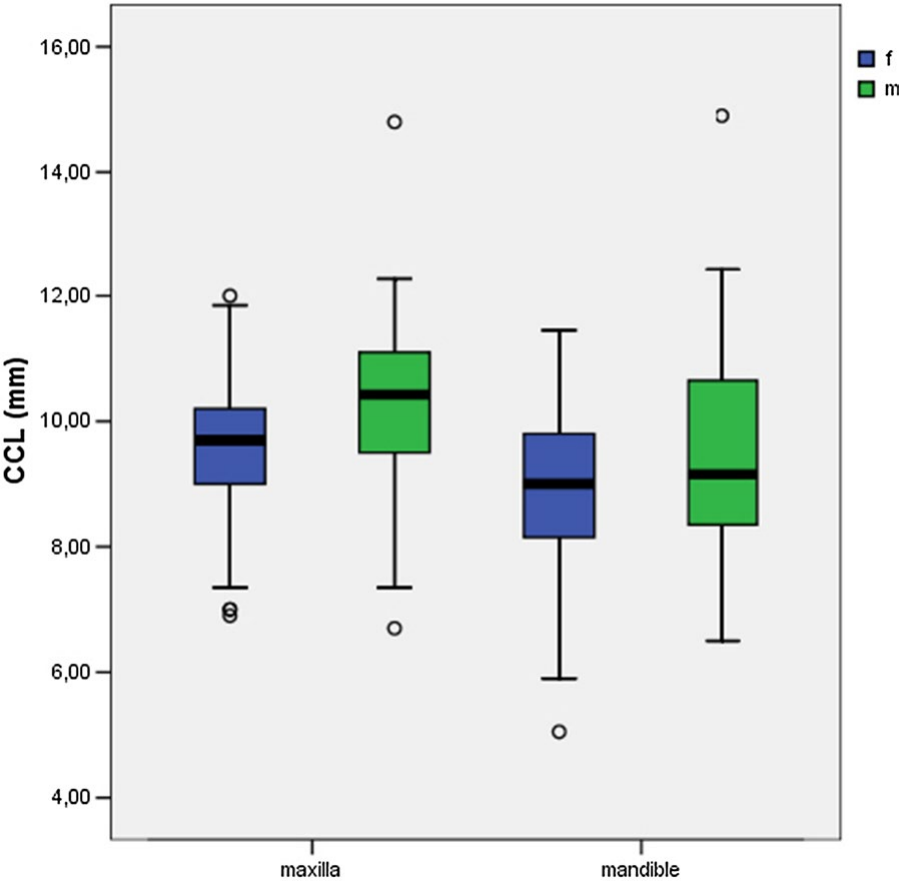
There was a significant difference in the clinical crown length (CCL) between males and females participants for the mandible (*P* ≤ 0.05) and maxilla (*P* ≤ 0.01). Male participants had significantly longer teeth than female participants

**Fig. 4:**
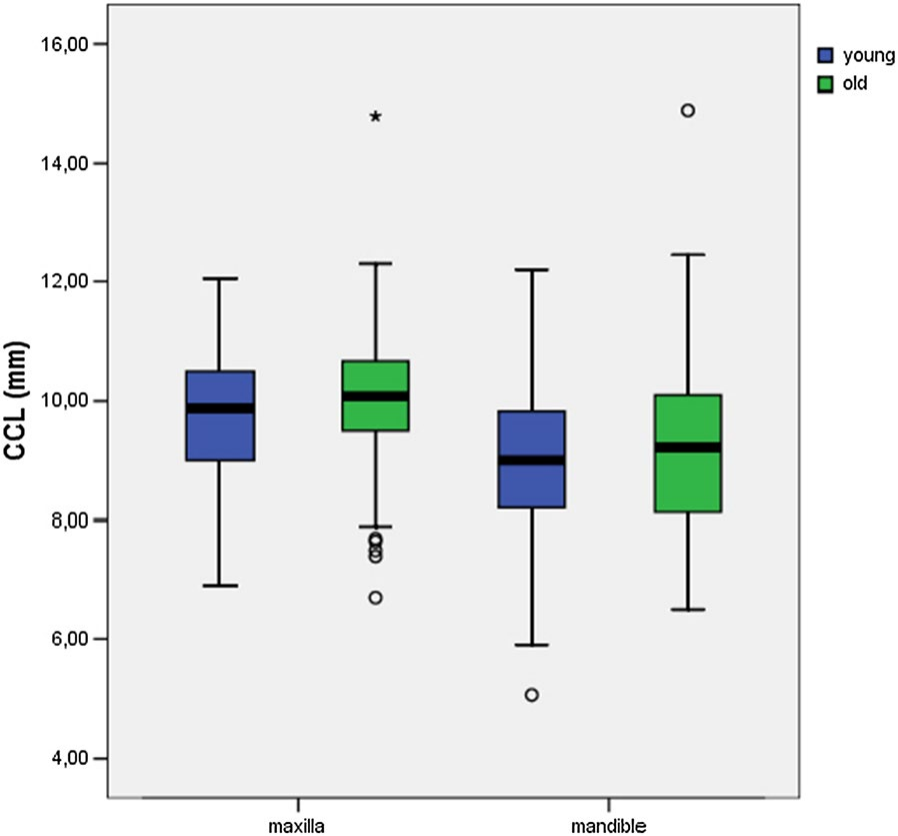
There were no significant differences between age groups with regard to the clinical crown length (CCL) of the upper (*P* = 0.06) and lower jaw (*P* = 0.33)

There was a significant difference in CCL between male and female participants for the mandible (*P* ≤ 0.05) and maxilla (*P* ≤ 0.01) (Table 3). Male participants had significantly longer teeth than female participants (Fig. 3). No significant differences were found between age groups with regard to the CCL of the upper (*P* = 0.06) and lower jaw (*P* = 0.33) (Fig. 4).

**Table 3:** Statistical differences were documented for maxillary and mandibular teeth between the genders regarding the CCL

Clinical crown length	
Gender (f/m)	Age (y/o)
*P* value	*P* value
*Maxilla*	
< 0.01	0.06
*Mandible*	
< 0.05	0.33

Width of attached gingiva	
Sex (f/m)	Age (y/o)
*P* value	*P* value
*Maxilla*	
0.69	< 0.01
*Mandible*	
0.26	< 0.05

Between the age groups, no statistical differences could be determined for the CCL. Regarding the WAG, statistical differences were documented for maxillary and mandibular teeth between the age groups. Between the genders, no statistical differences could be determined

The CCL for incisors and canines differed significantly in the maxilla (*P* ≤ 0.05) and mandible (*P* ≤ 0.01). The CCL of incisors was higher in maxillary teeth, whereas in mandibular teeth the canines displayed higher values.

### WAG

The average WAG of maxillary teeth was 3.9 mm (SD = 1.32 mm) in incisors and 3.7 mm (SD = 1.22 mm) in canines. In the mandibular teeth, an average WAG of 2.6 mm (SD = 1.11 mm) was documented for the incisors, and 2.3 mm (SD = 0.80 mm) was documented for the canines. The distribution of the WAG with regard to age and gender is displayed in Table 2.

**Fig. 5:**
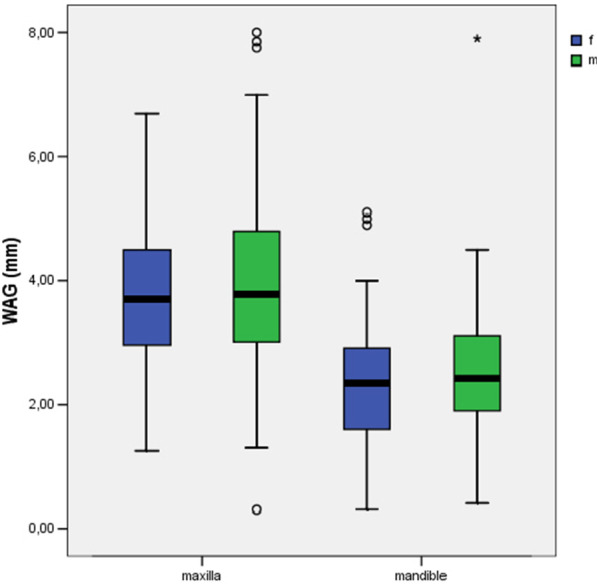
There were no significant differences in width of attached gingiva (WAG) between male and female participants for the mandible (*P* = 0.26) and maxilla (*P* = 0.69)

**Fig. 6:**
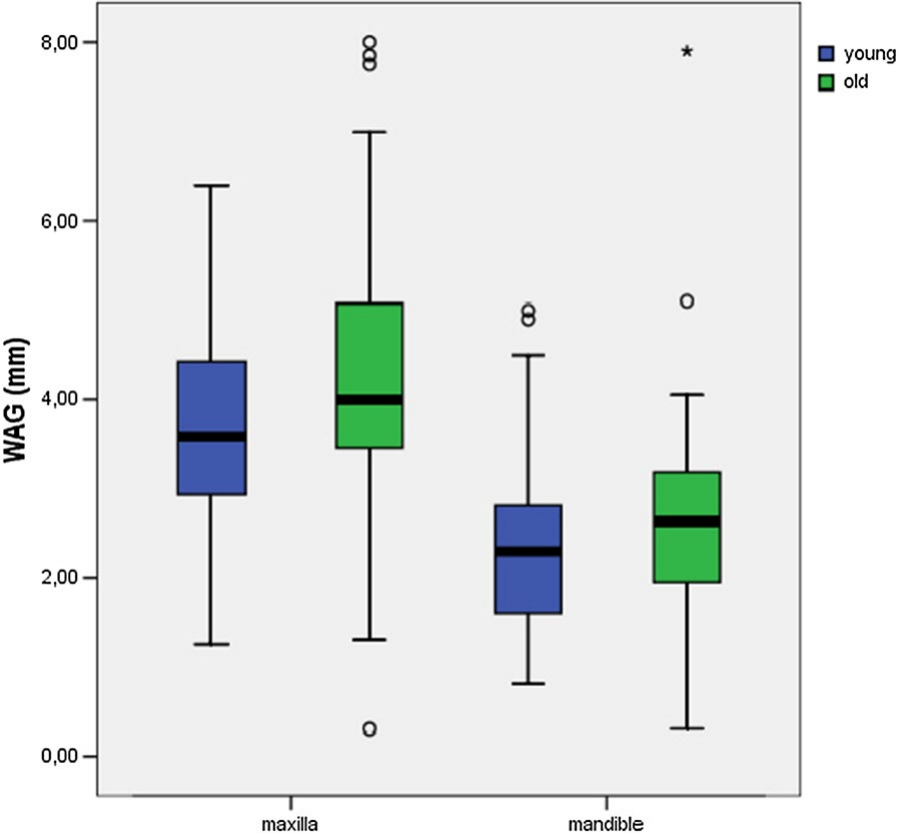
There was a significant difference in width of attached gingiva (WAG) between young and old participants for the maxilla (*P* ≤ 0.01) and mandible (*P* ≤ 0.05)

There were no maxillary (*P* = 0.69) or mandibular (*P* = 0.26) differences in the WAG between genders (Table 3, Fig. 5). Significant differences were found for the WAG of maxillary (*P* ≤ 0.01) and mandibular teeth (*P* ≤ 0.05) when comparing the young and old cohorts (Table 3, Fig. 6), with the attached gingiva being wider in the older age group.

Furthermore, differences were found between the incisors and canines of the maxilla (*P* ≤ 0.05) and mandible (*P* ≤ 0.01) (Table 4), with the WAG being higher on maxillary and mandibular incisors than on canines.

**Table 4:** Statistical differences between CCL and WAG of maxillary and mandibular incisors and canines were documented

	Incisors/canines
	*P* value
*Teeth position and CCL*	
Maxilla	**< 0.01**
Mandible	**< 0.01**
*Teeth position and WAG*	
Maxilla	**< 0.05**
Mandible	**< 0.01**

All significances are written in bold

### Relationship between CCL and WAG

The ratio between CCL and WAG was calculated using the quotient of CCL and WAG over all tooth positions. Subsequently, the differences between the ratios in the maxilla and mandible were determined using the Mann–Whitney *U* Test.

The quotient between CCL and WAG in the maxilla amounted to 3.10 (SD = 3.30) and 4.67 (SD = 3.68) in the mandible. This resulted in a ratio of 1:3 in the maxilla and 1:4.5 in the mandible. The ratio of CCL and WAG differed significantly between maxilla and mandible (*P* ≤ 0.01).

There were gender-related differences in the CCL (*P* ≤ 0.01) and the WAG (*P* ≤ 0.01) between the maxillary and mandibular teeth. Both the CCL and the WAG were higher in the maxilla than in the mandible.

To ascertain whether gender, age, and position have an influence on the CCL and WAG, a nonparametric analysis of variance was carried out (Table 5). It was shown, that gender (*P* ≤ 0.01) and position (*P* ≤ 0.01), but not age (*P* = 0.08), significantly influenced the CCL in both jaws.

**Table 5:** The nonparametric analysis of variance showed that gender and position had a significant influence on the CCL of maxillary and mandibular teeth

Nonparametric analysis of variance			
	Sex	Age	Position
	*P* value	*P* value	*P* value
CCL of Maxilla	**< 0.01**	0.08	**< 0.01**
CCL of Mandible	**< 0.01**	0.18	**< 0.01**
WAG of Maxilla	0.88	0.052	**< 0.01**
WAG of Mandible	0.26	**< 0.05**	**< 0.05**

Regarding the WAG the position was shown to have a significant influence on the maxilla and mandible. In addition, the age influenced the WAG of the mandibular teeth

All significances are written in bold

In the maxilla, there was a significant relationship between the position and WAG (*P* ≤ 0.01), but not regarding the age (*P* = 0.052). For mandibular teeth, both age (*P* ≤ 0.05) and position (*P* ≤ 0.05) had a significant influence. Gender had no impact on the WAG in both jaws.

## Discussion

The present findings demonstrate that there is no correlation between the CCL and WAG in a healthy periodontium, meaning that physiologically shorter teeth do not correlate with less or more attached gingiva. However, gender, age, and position influence CCL and WAG.

Gender appeared to have an influence on the CCL, with Caucasian males having significantly longer teeth than females. The same results were reported by Yuan et al. [21] for an Indonesian population and by Choi et al. [22] for a Korean population with an average age of 27.2 ± 7.7 years. Similar results were reported by Morrow et al. [16] for 456 maxillary anterior teeth in Welsh probands aged between 11 and 12 years and 18 and 19 years. Yuan et al. and Morrow et al. measured the CCL with calipers, whereas Choi et al. analyzed the CCL using cone beam computed tomography. According to the author’s research, to date, no other studies in a Caucasian population are available. The above mentioned results indicate that the CCL is less influenced by origin than by gender.

Regarding age, the mean values of CCL in the present study tended to be higher in older than in younger participants, but without statistical significance. Similar results were found by Volchansky and Cleaton-Jones [17], who published a review of clinical crown heights in the human permanent dentition that included 11 published papers all using calipers and landmarks when collecting data. All studies except one [23] reported clinical crown heights at ages ranging from 7 to 20 years. The results revealed a significant increase in the clinical crown height of the central and lateral incisors with age. Similarly, Bassey et al. [23] measured 2048 anterior teeth of adult Nigerians and also documented an increase in clinical crown height with increased age. Furthermore, they concluded that Nigerians have shorter crowns than Caucasians.

Choi et al. [22] examined the CCL of a Korean population in relation to age (n = 672), in a sample with a mean age of 27.2 ± 7.7 years, and found an increase in the root-crown ratio of the mandibular incisors with age. It seems that the Korean population has shorter teeth than Nigerians and Caucasians. It should be noted that no studies on CCL, that included participants in older age groups have yet been carried out. However, the variation in tooth size in different populations has already been documented by Hanihara and Ishida [24]. After evaluating the mesiodistal and buccolingual crown diameter of 72 major human population groups, the authors concluded that Australians have the largest teeth, whereas Western Europeans have small teeth. East/Southeast Asians are intermediate in overall tooth size [24]. Furthermore, meaningful comparable data in different populations analyzing the crown height are lacking.

Regarding the WAG, there was no significant influence of gender in the present study, but there were high inter-individual variances. In this context, a few probands showed a WAG of only 0.3 mm, whereas others showed a WAG of up to 8.0 mm. Reasons for the interindividual variance are yet unexplained. These results were consistent with those of Kolte et al. [14], who found no differences in WAG between males and females in an Indian population. Kolte et al. included 3 different age groups (16–24 years, 25–39 years, and > 40 years; n = 20 males, 20 females in each group) and reported smaller mean amounts of attached gingiva in the maxilla compared to the present study. Adesola et al. [25] also investigated the influence of gender on the WAG of 54 females and 19 males in a Nigerian population and found no significant differences either. Contrary to the findings of Adesola et al., Shaju and Zade [15] reported a higher WAG in Indian females than in males. It should be noted that the authors analyzed the measurements on the maxillary and mandibular central incisors, the premolars, and molars, but they did not present gender-related positional details. The mean amount of attached gingiva of the maxillary and mandibular central incisor was comparable to that reported in the current study.

A correlation between the WAG and age was identified, which is consistent with the findings of Ainamo et al. [13], who documented an increasing WAG with higher age. Srivastava et al. [26] also examined the change in WAG with age in Indian children between the ages of 4 and 15 years and found an increasing WAG with age and a concomitant reduction of sulcus depth in permanent teeth. Similar outcomes were reported by Kolte et al. [14], who found highly significant differences between the WAG in different age groups of an Indian population. A possible explanation for an increasing WAG could be the continuous coronal shift of the cementoenamel junction over the course of adult life, which can be attributed to continuous eruption of the teeth to compensate for natural tooth wear [12]. This explanation would imply that the origin of the proband has no influence on the WAG since natural tooth wear occurs in every population. However, this has not yet been addressed in the literature; although, it is assumed that geographic areas and cultures may influence tooth wear lesions [27].

Regarding the tooth position, there was a significant influence on CCL and WAG in both the maxilla and mandible in the present study. The WAG on incisors was significantly higher than on canines. Clinically longer crowns did not have an influence on WAG. Similar results were reported by Ainamo and Löe [11], who documented the highest WAG on the maxillary and mandibular incisors of Danish probands. The WAG in both jaws decreased towards the molar regions, and the narrowest zone was next to inserting frena and muscle attachments. In a study of the WAG of all permanent teeth of 100 Iranian dental students between the ages of 20 and 24 years, the highest values were reported for the lateral incisors; although, there was no statistical analysis [28]. Besides variations of the tooth size in different populations, there are also variations of WAG but further studies with larger cohorts are needed.

In conclusion, the mean values of cohorts with mixed age and gender groups and cohorts of different origins should not taken into consideration when attempting to determine the optimum relationship between the pink and white aesthetics. This is because there are age- and gender-related differences in the anatomy of the oral cavity. In addition, the position of the teeth and the origins of the populations studied should be considered when planning prosthetic reconstructions since differences in the WAG and CCL have been reported in the literature.

Since the CCL does not appear to have any influence on the WAG, the length of reconstructed crowns should not have any reverse effect on the WAG. However, as this was the first study on the relationship between the CCL and WAG of natural teeth, further research on this topic should be undertaken.

## Conclusion

There is no correlation between the CCL and the WAG in a healthy periodontium. Gender influences the CCL, with men having significantly longer teeth than women. Age has no significant influence on the CCL. Gender has no significant influence on the WAG. Age has a significant influence on the WAG.

## Data Availability

The datasets used and/or analyzed during the current study are available from the corresponding author on reasonable request.

## Abbreviations

CCL: Clinical crown length
WAG: Width of attached gingiva
MGJ: Mucogingival junction
